# Epidemiology, Impact and Control of Rabies in Nepal: A Systematic Review

**DOI:** 10.1371/journal.pntd.0004461

**Published:** 2016-02-12

**Authors:** Brecht Devleesschauwer, Arjun Aryal, Barun Kumar Sharma, Anita Ale, Anne Declercq, Stephanie Depraz, Tara Nath Gaire, Gyanendra Gongal, Surendra Karki, Basu Dev Pandey, Sher Bahadur Pun, Luc Duchateau, Pierre Dorny, Niko Speybroeck

**Affiliations:** 1 Department of Virology, Parasitology and Immunology, Faculty of Veterinary Medicine, Ghent University, Ghent, Belgium; 2 Institute of Health and Society (IRSS), Université catholique de Louvain, Brussels, Belgium; 3 Emerging Pathogens Institute and Department of Animal Sciences, University of Florida, Gainesville, Florida, United States of America; 4 Central Veterinary Hospital, Ministry of Agricultural Development, Kathmandu, Nepal; 5 Department of Livestock Services, Ministry of Agricultural Development, Kathmandu, Nepal; 6 Institute of Veterinary, Animal and Biomedical Sciences, Massey University, Palmerston North, New Zealand; 7 National Zoonoses and Food Hygiene Research Centre, Kathmandu, Nepal; 8 Laboratory of Experimental Hematology, Vaccine and Infectious Disease Institute (Vaxinfectio), Faculty of Medicine and Health Sciences, University of Antwerp, Edegem, Belgium; 9 Unité Mixte de Recherche - Contrôle des Maladies Animales, Exotiques et Émergentes (UMR CMAEE), CIRAD, Petit-Bourg, Guadeloupe, France; 10 Disease Surveillance and Epidemiology, WHO Regional Office for South East Asia, New Delhi, India; 11 Department of Pathobiology, College of Veterinary Medicine, University of Illinois, Urbana-Champaign, Illinois, United States of America; 12 Leprosy Control Division, Department of Health Services, Ministry of Health and Population, Kathmandu, Nepal; 13 Clinical Research Unit, Sukraraj Tropical & Infectious Disease Hospital, Teku, Kathmandu, Nepal; 14 Department of Comparative Physiology and Biometrics, Faculty of Veterinary Medicine, Ghent University, Ghent, Belgium; 15 Department of Biomedical Sciences, Institute of Tropical Medicine, Antwerp, Belgium; American Heart Association, Inc, UNITED STATES

## Abstract

**Background:**

Rabies is a vaccine-preventable viral zoonosis belonging to the group of neglected tropical diseases. Exposure to a rabid animal may result in a fatal acute encephalitis if effective post-exposure prophylaxis is not provided. Rabies occurs worldwide, but its burden is disproportionately high in developing countries, including Nepal. We aimed to summarize current knowledge on the epidemiology, impact and control of rabies in Nepal.

**Methods:**

We performed a systematic review of international and national scientific literature and searched grey literature through the World Health Organization Digital Library and the library of the National Zoonoses and Food Hygiene Research Centre, Nepal, and through searching Google and Google Scholar. Further data on animal and human rabies were obtained from the relevant Nepalese government agencies. Finally, we surveyed the archives of a Nepalese daily to obtain qualitative information on rabies in Nepal.

**Findings:**

So far, only little original research has been conducted on the epidemiology and impact of rabies in Nepal. Per year, rabies is reported to kill about 100 livestock and 10–100 humans, while about 1,000 livestock and 35,000 humans are reported to receive rabies post-exposure prophylaxis. However, these estimates are very likely to be serious underestimations of the true rabies burden. Significant progress has been made in the production of cell culture-based anti-rabies vaccine and rabies immunoglobulin, but availability and supply remain a matter of concern, especially in remote areas. Different state and non-state actors have initiated rabies control activities over the years, but efforts typically remained focalized, of short duration and not harmonized. Communication and coordination between veterinary and human health authorities is limited at present, further complicating rabies control in Nepal. Important research gaps include the reporting biases for both human and animal rabies, the ecology of stray dog populations and the true contribution of the sylvatic cycle.

**Interpretation:**

Better data are needed to unravel the true burden of animal and human rabies. More collaboration, both within the country and within the region, is needed to control rabies. To achieve these goals, high level political commitment is essential. We therefore propose to make rabies the model zoonosis for successful control in Nepal.

## Author Summary

Rabies has been known as a deadly disease in Nepal for decades, but information on epidemiology, impact and control remains scattered. We collected and summarized information from a variety of sources, including scientific literature and government agencies. Only little original research has been conducted on the epidemiology and impact of rabies in Nepal, leaving many questions unanswered. Official reports show that each year 100 livestock and 10–100 humans die of rabies, but these numbers very likely underestimate the true rabies burden. Availability and supply of anti-rabies vaccines have remained suboptimal and rabies control activities have been hampered by a lack of collaboration and comprehensiveness. High level political commitment is essential to overcome these problems and to reduce the burden of rabies. We therefore propose to make rabies the model zoonosis for successful control in Nepal.

## Introduction

Rabies is a neglected zoonotic disease caused by an RNA virus of the family *Rhabdoviridae*, genus *Lyssavirus*. All mammals can be infected with the rabies virus, but dogs are the most important source of human rabies. Although the necessary evidence and tools are in place to control and eliminate rabies, the virus still has a worldwide distribution and is causing a significant health and economic burden to mainly developing countries in Africa and Asia [1].

Rabies is a vaccine-preventable disease. Modern cell culture-based and embryonated egg-based anti-rabies vaccines (ARV) have proven to be safe and effective in preventing human rabies [2]. Earlier nerve tissue ARV induce severe adverse reactions and are less immunogenic. As a result, the production and use of nerve tissue ARV has been discouraged by the World Health Organization (WHO) since 1984, although they are still in use in a few countries [3]. Pre-exposure prophylaxis (PrEP) is recommended for individuals who will be at continual, frequent or increased risk of exposure to the rabies virus [2], such as animal handlers, laboratory technicians, and veterinarians in endemic countries. Once exposed to a rabid animal, timely post-exposure prophylaxis (PEP) can be lifesaving. The WHO-recommended PEP protocol consists of immediate and proper primary wound management, accompanied by a recommended course of ARV and, for high risk exposures, administration of rabies immunoglobulin (RIG). Intradermal administration of ARV is recommended over intramuscular administration, as it reduces the volume used and thus the direct cost of the vaccine by 60–80%, without compromising on safety or immunogenicity [3].

Although proper implementation of PrEP and PEP can significantly reduce the human rabies burden, it is neither a sustainable nor a cost-effective approach for controlling rabies. Indeed, human rabies prophylaxis alone does not reduce the rabies transmission and can induce an unbearable economic burden on households, communities and governments. The *Partners for Rabies Prevention*, an international group of agencies and experts involved in rabies, developed a blueprint for rabies prevention and control [4]. The main intervention strategy in the dog rabies control blueprint is mass dog vaccination, possibly complemented with dog population management measures. However, proper planning and evaluation are equally crucial components of the blueprint. In the planning phase, information should be gathered on the local rabies epidemiology and the extent of the dog population. Also, awareness should be created and support elicited from both the local population and the relevant governmental agencies. Once a programme is in place, the change in epidemiological, economic and social impact of the disease needs to be monitored to evaluate the effectiveness of the programme. Reliable baseline data and effective rabies surveillance are inevitable to accomplish this goal.

This review focuses on the rabies situation in Nepal. Landlocked between India and China, Nepal has a population of approximately 28 million and a surface of 147,000 km², administratively divided in 75 districts. Geographically, the country can be divided in three ecological belts, i.e., the northern Himalayas, the central hills, and the southern Terai plains. Due to a concurrent history and an open border, Nepal has similar socioeconomic conditions as India, the country with the largest rabies burden worldwide [1,5]. Nevertheless, little is known about the actual status of rabies in Nepal. This review summarizes current knowledge on epidemiology, impact and control of rabies in Nepal, and ends with recommendations for a way forward.

## Materials and Methods

We used a variety of sources to search for information on the epidemiology, impact and control of rabies in Nepal (S1 and S2 Files). Rabies epidemiology was defined as transmission, geographical distribution, seasonality and molecular diversity, while rabies impact was defined as the number of outbreaks, cases, deaths and Disability-Adjusted Life Years (DALYs). We performed a systematic review of scientific literature indexed in PubMed, Web of Knowledge and Nepal Journals Online (http://www.nepjol.info/), complemented by manual searches of the main Nepalese journals and the conference proceedings of the Rabies in Asia (RIA) foundation (http://www.rabiesinasia.org/). We searched for the following key words: ("rabies" OR "rabid" OR "dog") AND "Nepal". After removing duplicates, we first excluded items for which we could not retrieve an abstract or full text, and subsequently excluded items that did not pertain to rabies in Nepal. No time restrictions were applied. Further grey literature was collected through searching the WHO Digital Library (http://apps.who.int/iris/) and the library of the National Zoonoses and Food Hygiene Research Centre (NZFHRC), a non-governmental organization actively working in the prevention of zoonosis in Nepal. We also searched Google and Google Scholar for additional documents, but acknowledge that these searches are not replicable, due to the continuous updating of the Google databases and the user-specific ranking of database items. For each eligible document, a narrative synthesis was made, which were then further digested into a qualitative review.

Additionally, we obtained data on animal and human rabies from the relevant Nepalese government agencies, and generated numerical and graphical summaries by district, year and month. Rabies surveillance in Nepal is passive and based on decentralized data collection systems. The Veterinary Epidemiology Centre (VEC), resorting under the Directorate of Animal Health (DAH), Department of Livestock Services (DLS), Ministry of Agricultural Development (MoAD), is the national focal point for animal disease surveillance, including rabies. Passive surveillance for bite incidents (due to dogs and other animals) is comprised in the Health Management Information System (HMIS), managed by the Department of Health Services (DoHS), Ministry of Health and Population (MoHP). The Epidemiology and Disease Control Division (EDCD) under the DoHS is responsible for prevention and control of rabies in Nepal, and for recording human rabies cases and PEP administration.

Finally, we searched news items on rabies in Nepal from the 2010–2014 archives of The Himalayan Times, a large English-language Nepalese daily. We acknowledge that this data source does not provide reliable quantitative information, but believe that it is a useful source of qualitative information on the rabies situation in Nepal.

## Results

### Data sources

**Fig 1:**
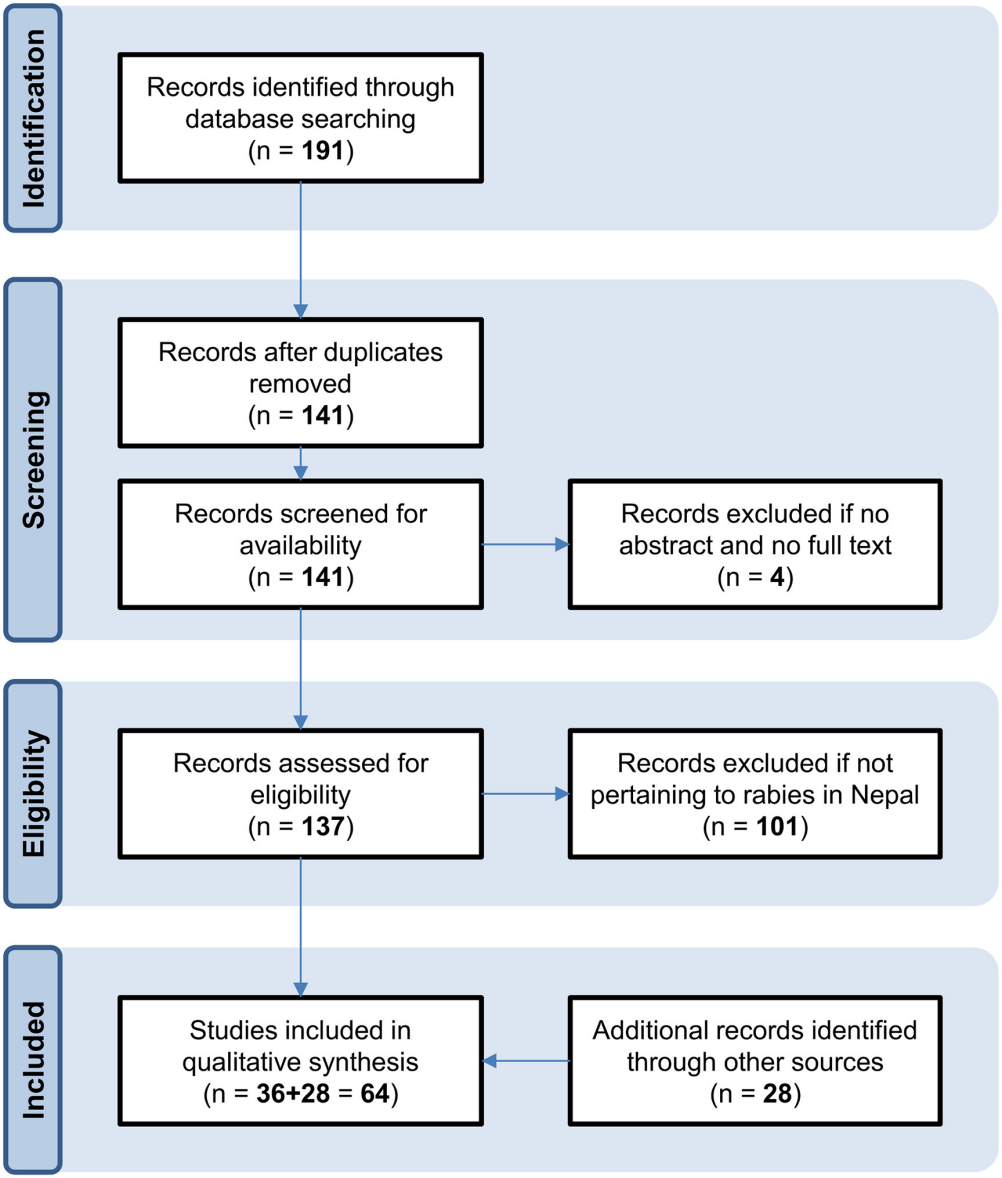
Flow diagram of systematic review.

Little original research has been conducted on the epidemiology and impact of rabies in Nepal. Fig 1 shows a flow diagram summarizing the results of the systematic review. From the database searches, we retained a total of 36 documents (S1 File), while the further manual searches revealed another 28 documents (S2 File). In the period 2010–2014, 41 articles were published about rabies in The Himalayan Times (S3 File).

We retrieved data from the VEC for the period 2005–2014. Similar data from previous time periods have been discussed by Gongal [6] and Karki and Thakuri [7]. The VEC receives a monthly *Animal Disease Epidemiological Report* in a specified format from all 75 District Livestock Service Offices (DLSO). Each DLSO, in its turn, receives animal health and disease data from 999 livestock service centres strategically located in the various Village Development Committees and Municipalities, the lowest administrative levels in the Nepalese system [6]. Reported cases are mostly based on clinical diagnosis without lab confirmation. In the absence of a standardized case definition, diagnosis of animal rabies depends on the clinical experience of the practitioner. Further information on rabies in Nepal was available from the five most recent DAH Annual Technical Reports [8–12].

Since 1994, the DoHS publishes *Annual Reports* which analyse the performance of different programmes and present information collected by the HMIS. In fiscal year 2013/14, 81% of public hospitals, all 75 District (Public) Health Offices, and all Primary Health Care Centres, Health Posts, and Sub Health Posts reported to HMIS [13]. A total of 441 NGO and 669 private health institutions also reported to HMIS that year. The Annual Report contains information on the number of dog bites and other animal bites, by district, reported to the concerned health centres. It also contains hospital inpatient data on rabies morbidity and mortality, based on the ICD codes A82 (Rabies) and A82.8 (Rabies, unspecified). We obtained the ten most recent DoHS Annual Reports, i.e., for fiscal years 2004/05 to 2013/14 [13–22].

### Epidemiology

Rabies in Nepal occurs in two interrelated epidemiological cycles: an urban cycle involving domesticated dogs and a sylvatic cycle involving wildlife [23]. The urban cycle is the predominant source of human rabies, with more than 96% of rabies patients reported during 1991–2000 showing a history of rabid dog exposure [24]. Nevertheless, overlap between both cycles does occur. Indeed, spill-over between cycles has recently been demonstrated by the isolation of a virus from a human rabies case that showed 100% identity over the studied region to viruses previously isolated from two dogs and a mongoose (family *Herpestidae*) in Nepal [25].

The urban cycle is maintained by the stray and community dog population, with spill-overs to pet dogs adding to the human rabies burden. There is little current information on the extent of the stray dog population in Nepal. Based on a dog census carried out by the NZFHRC in 1998, it was estimated that there were nearly 2 million dogs in Nepal at that time (or 1 per 10 humans; [26]. However, most other surveys have been conducted in the Kathmandu Valley of Nepal, comprising the Kathmandu, Bhaktapur and Lalitpur districts. In 1989, Bögel & Joshi [27] estimated a dog population of 12,500 in Lalitpur city, or 700 per km². In October 1997, a much higher stray dog density of 2,930 dogs per km² was established in Kathmandu [28], corresponding to over 170,000 stray dogs (assuming a total area of 58 km²). More recently, animal welfare organizations have undertaken several dog population surveys in the Kathmandu Valley. Within the Ring Road area of the Valley, the estimated dog population was 31,000 in 2006, dropping to 22,500 in 2010 and 22,300 in 2012 [29,30]. In Pokhara, a sub-metropolitan city in western Nepal, a total of 1767 street dogs (32 per km²) were counted during a three-month survey in 2011–2012 [31].

One cause of this problematic size is believed to be the religious adoration of the dog in Nepalese culture [27]. The culmination of dog worship takes place on *Kukur Tihar*, the second day of *Tihar*, the festival of lights, when dogs receive religious ornaments and food. However, more important factors for the sustenance of the stray dog population are probably the bad garbage policy and open slaughter facilities, especially in the Kathmandu Valley [32,33]. The rapid urbanization and the growth of slum areas further create favourable conditions for the sustenance of stray dog populations [34,35]. Finally, the decline in the vulture population since the 1990s in the Indian subcontinent, including Nepal, also implied the loss of a competitor for food [36].

The sylvatic cycle is maintained by wild carnivores living in forest zones, national parks, or wildlife reserves, such as mongooses (family *Herpestidae*) and jackals (*Canis aureus*) [24]. In Nepal, the direct importance of this cycle is thought to be less important, although it probably has a significant indirect importance as continuous source of infection for the urban cycle [23,25]. Nevertheless, a proper understanding of the sylvatic cycle is lacking.

Rhesus macaques (*Macaca mulatta*) are abundant in certain temple areas inside and outside of the Kathmandu Valley. Although these temple monkeys can become infected through the urban and sylvatic cycle, their role in rabies transmission is unclear. Nevertheless, monkey bites or scratches are reported to occur frequently in tourists and expats staying in Kathmandu [37–40], and in India, a rhesus macaque is believed to have transmitted rabies to a 10-year-old Australian boy [38,41]. Furthermore, monkeys are occasionally reported to menace the local population, although it is not always clear if this is due to rabies infection (S3 File).

**Fig 2:**
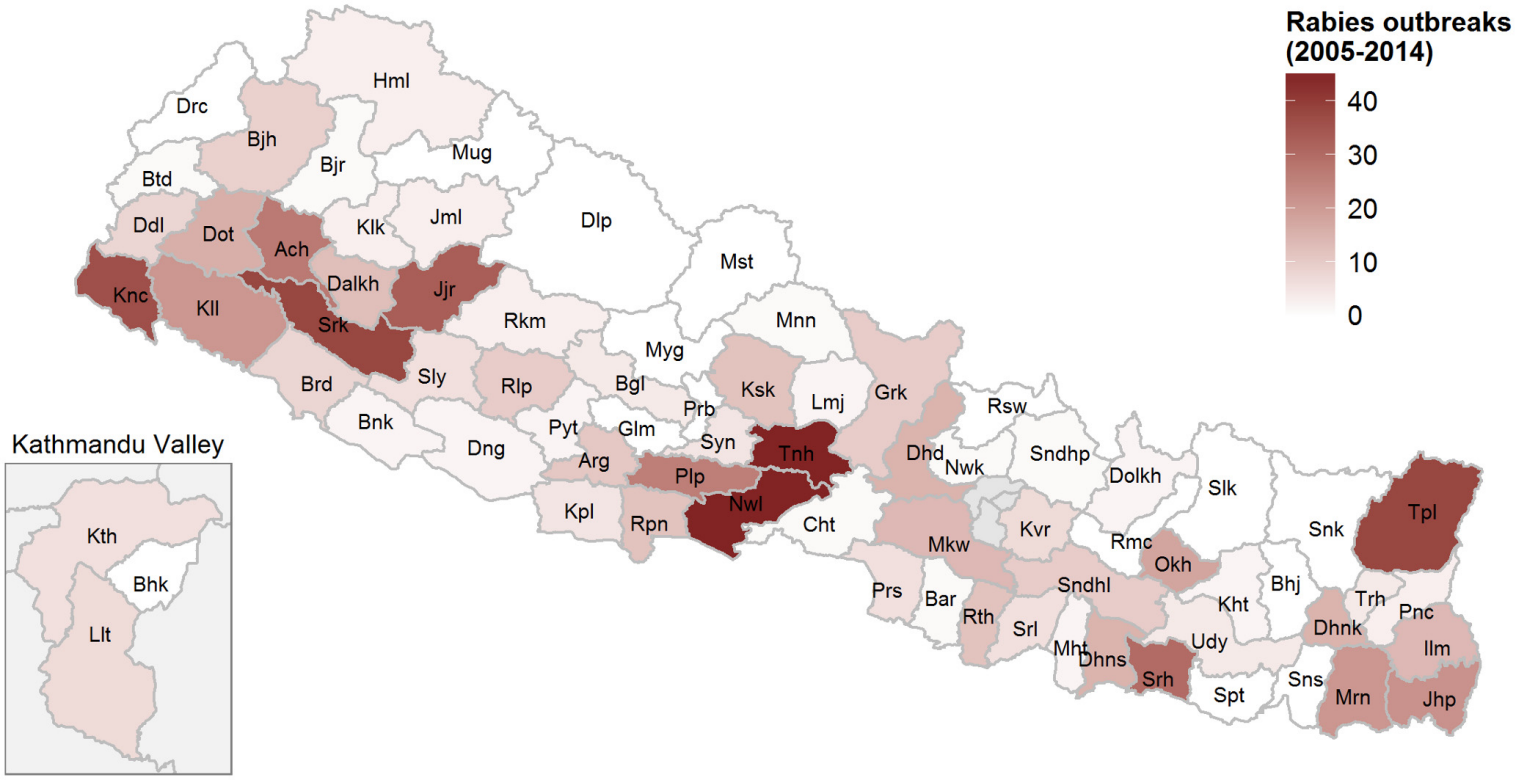
Number of rabies outbreaks reported during 2005–2014 to the Veterinary Epidemiology Center of the Directorate of Animal Health, Department of Livestock Services, Ministry of Agricultural Development. See S4 File for raw data and abbreviations.

The risk of rabies infection is believed to be highest in the southern Terai plains, which are densely populated agricultural areas and contain various wildlife areas [6,24]. The open border with India may also allow for spill-over between both countries. Nevertheless, animal and human rabies is reported in a much wider range of districts. Fig 2 shows the distribution of animal rabies outbreaks reported to the VEC during 2005–2014. The districts with the highest number of outbreaks were Nawalparasi and Tanahu (45 each). No outbreaks were reported from several mountain and hill districts during this period.

**Fig 3:**
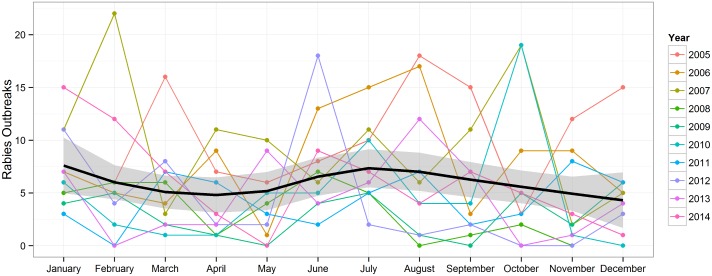
Monthly number of rabies outbreaks reported during 2005–2014 to the Veterinary Epidemiology Center of the Directorate of Animal Health, Department of Livestock Services, Ministry of Agricultural Development. The thick black line and grey ribbon represent the loess-smoothed mean and associated uncertainty interval. See S4 File for raw data.

Fig 3 shows the number of reported outbreaks by month. Over the ten-year period, the average monthly number of reported outbreaks ranged from 3.8 (November) to 7.6 (June and July). Some authors associated the apparent seasonality with the breeding seasonality of dogs and wild carnivores [6,7].

Molecular identification and phylogenetic analysis of animal rabies virus has identified both the Indian subcontinent lineage as the Arctic lineage to occur in animals in Nepal [42–44]. So far, only one wildlife sample has been analysed, i.e., from a mongoose, which was found to cluster with dog and livestock isolates [25,44]. No systematic monitoring for sylvatic rabies is in place.

### Impact

#### Animal rabies

Thakuri et al. [45] observed 34 rabies cases in cattle during 1985–1990 at the veterinary hospitals of four hill districts in Eastern Nepal. The only recent epidemiological data on animal rabies in Nepal stem from the VEC. The annual number of rabies outbreaks (defined as one or more animals with rabies symptoms) decreased from 121 in 2005 to 73 in 2013. During this period, a total of 1298 animals were reported dead and 152,400 vaccinated (Table 1). Cattle and buffalo appeared to be the most affected livestock species. Wildlife and primates were not covered by the VEC reporting system.

**Table 1:** Animal rabies cases and number of vaccinated animals during 2005–2014, as reported to the Veterinary Epidemiology Center of the Directorate of Animal Health, Department of Livestock Services, Ministry of Agricultural Development.

Species	Cases	Vaccinated
Dog	374	141,303
Cattle	442	4498
Buffalo	315	2857
Goat	122	2424
Sheep	9	587
Horse	14	288
Yak	1	252
Pig	21	191
**TOTAL**	**1298**	**152,400**

#### Human rabies

**Fig 4:**
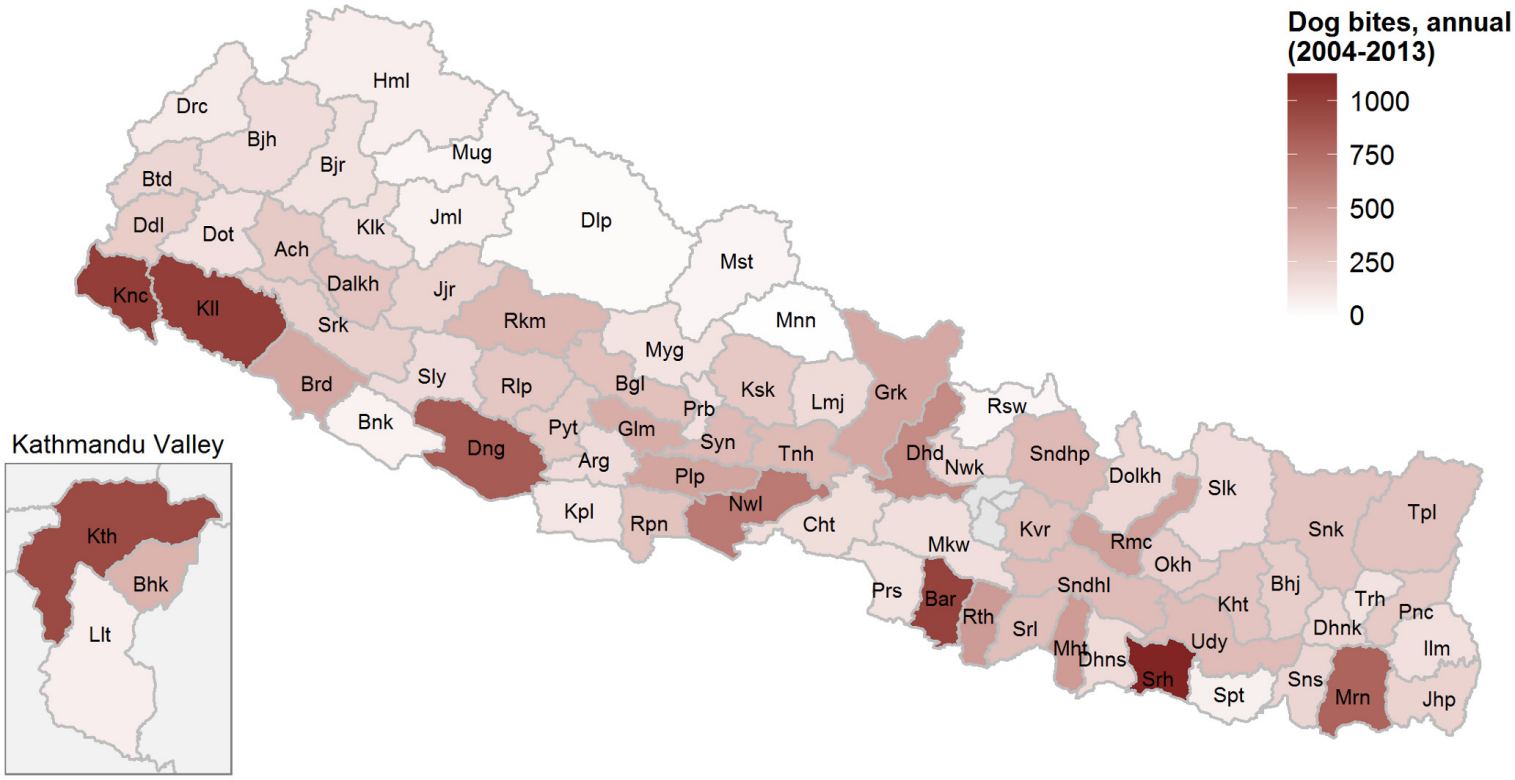
Median number of dog bites reported during 2004–2013 to the Department of Health Services, Ministry of Health and Population. See S4 File for raw data and abbreviations.

We identified two reports of travellers developing fatal rabies linked to dog bite incidents in Nepal (1970: Japan [46–48]; 1996: USA [49,50]). However, the most comprehensive source of epidemiological data on human rabies are the Annual Reports of the DoHS. The total number of reported dog bites showed a steady increase, from 15,000 in 2004 to 35,000 in 2013. Fig 4 shows the apparent distribution of dog bites in Nepal. Nearly 6% of all reported dog bites occurred in the districts of the Kathmandu Valley. The number of other animal bites stayed constant at around 2,000 per year. Inpatient morbidity cases (i.e., hospitalized cases) ranged between 1 and 28 per year, while inpatient mortality cases (i.e., hospitalized cases deceased while hospitalized) ranged between 0 and 6 per year. However, data from the EDCD showed that there were 97 deaths in 2008/09, dropping to 10 in 2013/14 [13].

**Fig 5:**
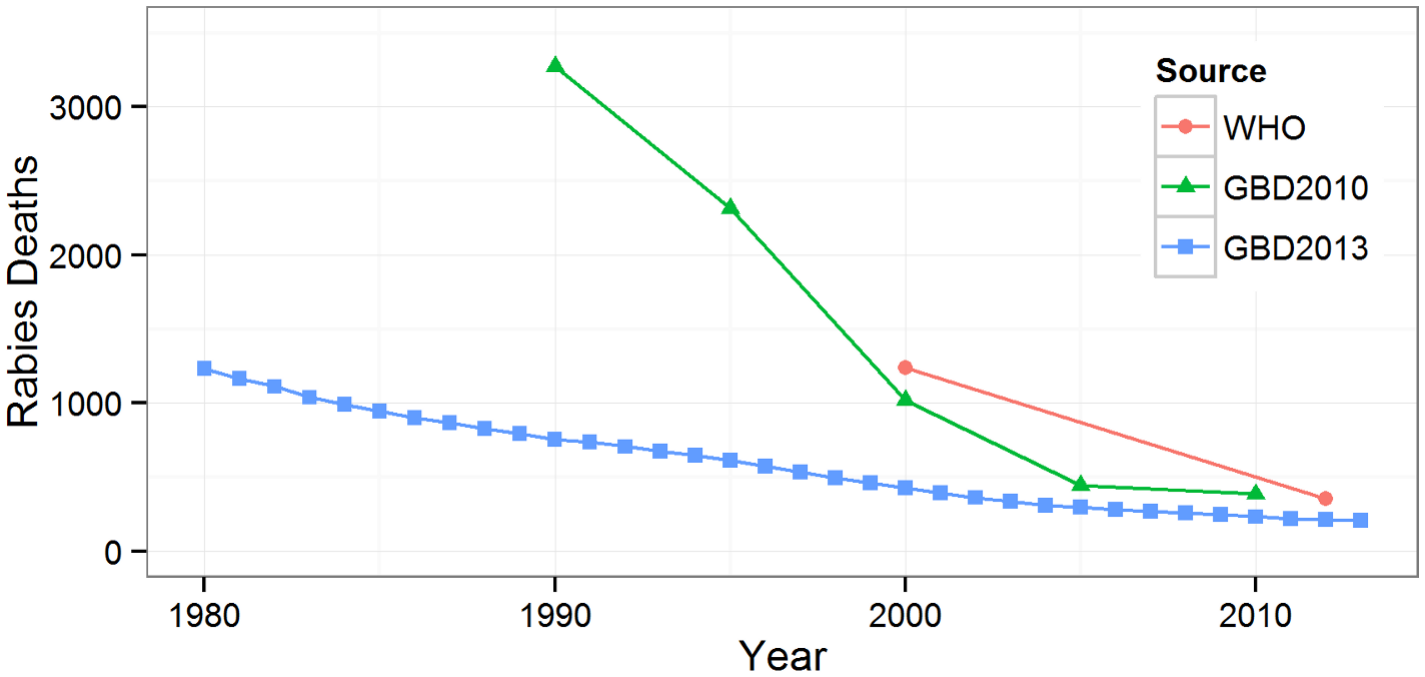
Estimated mean number of rabies deaths in Global Burden of Disease studies. WHO: World Health Organization Global Health Estimates [51]; GBD2010: Global Burden of Disease 2010 Study [52]; GBD2013: Global Burden of Disease 2013 Study [53].

Further information on the rabies burden in Nepal is available from the global burden of disease studies conducted by the WHO and the Institute for Health Metrics and Evaluation (IHME) (Fig 5). The available estimates indicate an important decrease in the annual number of rabies deaths, converging to 200–400 deaths in recent years [51–53]. The burden of rabies, expressed in DALYs, was estimated by WHO at 18,587 for the year 2012 [54] and by IHME at 12,200 for the year 2013 [55].

### Control

#### Prophylaxis

Production of ARV in Nepal started in 1970 and continues to date [56]. ARV production is managed by the Rabies Vaccine Production Laboratory (RVPL), which falls under the DAH, DLS, Ministry of Agricultural Development [8–12]. Distribution of ARV is handled via the five regional veterinary laboratories, which also serve as rabies vaccine banks to control outbreaks [57]. Initially, the RVPL produced phenolized 20% sheep brain ARV for pet immunization and phenolized 5% sheep brain ARV for post-exposure vaccination of livestock. In 1983/84, the RVPL started production of 5% Beta-Propiolactone (BPL) inactivated ARV of nerve tissue origin for human use, and was able to meet the national demand by 1994 [58,59]. Previously, ARV of nerve tissue origin was imported from India and vaccine scarcity was observed from time to time due to the vaccine’s short shelf life. Following the WHO recommendations, the production of nerve tissue ARV has now been phased out in favour of tissue or cell culture ARV. The veterinary authority phased out the production of phenolized 5% nerve tissue ARV in 2003 and that of phenolized 20% nerve tissue ARV in 2004 due to economical and ethical reasons [58]. The production of 5% BPL inactivated nerve tissue ARV for human use was phased out in 2006 [8–12].

To replace the nerve tissue vaccines, the RVPL worked with the Japan International Cooperation Agency (JICA) to set up cell culture ARV production facilities [60]; DAH 2008). A first trial batch of cell culture ARV for animal use was produced in 2002 [59], and after further development the first commercial batch was released in the market in 2006 under the trade name "NeJaRab". However, with a current target of 40–50,000 doses per year (which is not necessarily reached), the production of NeJaRab does not meet national demand, necessitating the import of additional ARV for animal use [57,61]. Also in 2006, a first trial batch of a Vero cell culture-based ARV for human use was produced, and in 2009, a first trial batch of hyper-immune serum from sheep was produced [59]. Today, the productions of cell culture ARV and RIG for human use are still reported to be in trial phase [61]. WHO is further encouraging Nepal to introduce the more cost-effective intradermal rabies vaccination schedule to improve and sustain accessibility and affordability of PEP [62].

The Government of Nepal is providing free ARV for human use at government hospitals and health centres since 2007 [58,63]. Despite the progress made in ARV production capacity, Nepal is 100% dependent on import for covering its PrEP and PEP need. For the three-year period 2013–15, the Government of Nepal with support from the World Bank purchased 900,000 ARV vials from an Indian manufacturer, at a total cost of 220 million NRs (1.8 million Euro). This would be sufficient to provide PEP to 30–40,000 people per year [13]. Nevertheless, media reports show that the availability and supply of government-provided ARV is sometimes insufficient, in the best case forcing people to resort to the private market for more expensive vaccines or, in the worst case, depriving people of PEP (S4 File). Furthermore, equine or human RIG is available only in clinics in Kathmandu, and due to the high cost, mainly used by tourists and expats [24,64].

#### Rabies control programmes

Over the past decades, various state and non-state actors have been involved in rabies control activities in Nepal. The driving force behind many of these projects appears to have been Dr Durga Datt Joshi, who served various government positions in the health and agriculture sector, including that of Chief Zoonotic Diseases Control Section. In 1989, he founded the NZFHRC, a research-oriented NGO [65], which he chaired until his demise in 2013. In recent years, various animal welfare organizations have started engaging in dog population management and rabies control activities. The most notable such organization is the Kathmandu Animal Treatment (KAT) Centre, which has been active in animal birth control and dog rabies vaccination in Kathmandu since 2004. Other organizations include Animal Nepal, Himalayan Animal Rescue Trust, Bhaktapur Animal Welfare Society, Society for the Prevention of Cruelty to Animals Nepal, and the various Kennel Clubs that have been set up throughout the country.

The first reference to a rabies control programme in Nepal was given by Bahmanyar [66]. In 1979, a national coordinating committee for dog rabies elimination was established, composed of different government representatives. However, it was not until 1983 that the first rabies control programme was implemented, supported by the Department of Livestock Development and Animal Health (DLDAH), the Department of Health, and representatives of local government [66]. A door-to-door survey prior to the campaign showed that 96% of pet dog owners showed willingness to participate but one in ten would object to a project removing stray dogs from their area. The actual programme involved creating awareness and setting up vaccination posts, eventually leading to the vaccination of 22,334 pet dogs in the Kathmandu Valley (and 14,472 in other districts by distributing surplus vaccine to district veterinary hospitals). These results were presented in November 1985 at the first national seminar on rabies, organized by Ministry of Health with support from WHO and UNDP [67]. During this seminar, recommendations were made for a national rabies control programme.

Also in the 1980s, Dr DD Joshi and Dr Konrad Bögel, then Chief of the WHO Veterinary Public Health unit, worked out plans for a four-year vaccination campaign. To support these plans, an *Intercountry Practical Training Course on Dog Population Management* was organized in 1986 in Kathmandu by the WHO Regional Office for South-East Asia [68], and several extensive dog population studies conducted in the late 1980s [27,69–71]. In June–July 1989, a first pilot project was conducted in Lalitpur City, during which around 8200 dogs were vaccinated [72]. Despite encouraging results, however, we could not retrieve any information pointing at a continuation of the project beyond the pilot phase.

In 2000–2008, the NZFHRC engaged in free dog rabies vaccination campaigns supported by the *Donative Unit for Rabies Vaccine to Nepal*, Tokyo, Japan. Mass dog vaccination and awareness programmes were organized in 24 municipalities across the country, in collaboration with the respective local governments. Over the eight-year period, a total of 18,973 dogs and cats were reported to be vaccinated [73–75].

Since 2001, faculty and students at the Cummings School of Veterinary Medicine, Tufts University, have been involved in rabies control activities in Nepal [76,77]. Partnerships have been set up with the NZFHRC, the KAT Centre, the Himalayan College of Agricultural Sciences and Technology (HICAST), and the Chitwan-based Institute of Agriculture and Animal Science (IAAS). Based on a workshop organized in April 2001 in Kathmandu, a concept for a *Nepal National Rabies Control Programme* was developed, aiming for the control of rabies in Nepal over a ten-year period [78]. Since 2002, however, Tufts’ efforts have been refocused on dog sterilization and rabies vaccination capacity building and student exchanges at IAAS and the KAT Centre (http://vet.tufts.edu/international-veterinary-medicine/projects-ivm/rabies-control-in-nepal/).

In April 2007, the NZFHRC organized a *Workshop for Consensus Building amongst National Alliance Partners to Eliminate Canine Rabies in Nepal and Development of the National Strategic Plan* [79]. Supported by WHO, the aim of the workshop was to develop a National Rabies Control Plan towards control of rabies by 2012 and elimination by 2027. Following the workshop, an *Alliance Group for Rabies Control in Nepal* was established in 2008, comprising the NZFHRC, the KAT Centre, the Veterinary Public Health Division of the DLS, and the Department of Public Health and Social Welfare of Kathmandu Metropolitan City. Since then, the Alliance Group has mainly been involved in dog vaccinations in Kathmandu, including the vaccination of 10,000 stray and community dogs in 2009 [12]. They are also involved in the annual promotion of World Rabies Day in Kathmandu, an initiative launched by the *Global Alliance for Rabies Control* (GARC) in 2007. On this day, September 28, free dog vaccination and awareness campaigns are organized. Currently, World Rabies Day is marked all across Nepal, with the support of various animal welfare and student organizations.

In 2013, the DLS received a donation of 200,000 doses of ARV from the World Organisation for Animal Health (OIE) [80]. However, this appeared to be a one-time support, with no reported follow-up activities.

Although the Government of Nepal has been involved in most of the aforementioned rabies control activities, it lacks a comprehensive and realistic national rabies control policy and strategy. There are currently no legal requirements regarding the registration and vaccination of animals, and rabies is not an officially reportable disease in Nepal. Moreover, until recently, the EDCD was still reported to supply free strychnine sulphate to local governments, leading to the elimination of around 20,000 stray dogs each year [73]. Communication between veterinary and human health authorities is limited when it comes to case reporting, outbreak investigation and control, which further complicates rabies control in Nepal.

Regional and international rabies control initiatives are emerging, which could help Nepal in its control efforts [81]. The institution of World Rabies Day by the GARC is just one example, but other initiatives are being launched by the WHO Regional Office for South East Asia [62,82], the South Asian Association for Regional Cooperation [23], and the RIA Foundation [81,83].

## Discussion

A proper understanding of the epidemiology and impact of rabies is crucial for planning, implementing and evaluating rabies control programmes. In this review, we have tried to generate the best possible summary of data on animal and human rabies in Nepal. However, as our review was mainly narrative in nature, and as a substantial amount of information was obtained through grey literature searches, we acknowledge that replicability may be limited. To accommodate this limitation, we ensured full transparency by including full details on our search strategy and results as Supporting Information files.

Per year, rabies is reported to kill about 100 livestock and 10–100 humans, while about 1,000 livestock and 35,000 humans are reported to receive rabies PEP. However, these estimates very likely represent serious underestimations of the true rabies burden. Indeed, underreporting is very likely to occur in both the animal and human passive surveillance systems [84], and a proper understanding and quantification of the various reporting biases is a major research gap. Illustrative for these problems are the discrepancies in human rabies deaths between the DoHS Annual Reports and the EDCD reports and the lack of inpatient data from three districts (Lalitpur, Parsa, and Rupandehi) since the last five years. Further perturbation is introduced by putative cases typically being diagnosed based on history and symptoms, without lab confirmation. Although rapid testing is done in regional laboratories, confirmation of rabies is currently limited to the Central Veterinary Laboratory, Kathmandu [42,61], which sees around 20 positive human and animal samples a year [8–12]. This corresponds to 10–20% of all reported cases, but to an unknown proportion of all cases. Underreporting of human rabies cases may further result from the fact that rabies patients sometimes prefer to visit traditional healers or prefer to stay home when rabies symptoms have appeared, resulting in a discrepancy between inpatient morbidity and inpatient mortality cases (S3 File). Human rabies cases may further be refused hospital admission due to fear of exposure to health workers and the absence of effective treatment. The reporting of animal rabies cases may depend on the economic value of the affected species, the remoteness of the area, and the motivation of the practitioner.

Rabies is estimated to cause around 10–20,000 DALYs per year in Nepal [54,55]. This is in line with the total burden of the three major parasitic zoonoses in Nepal (i.e., cysticercosis, toxoplasmosis, cystic echinococcosis; [85]), showing that rabies still is a major zoonosis in Nepal. However, in the absence of reliable data, the burden estimates generated by WHO and IHME are based on extrapolations from neighbouring countries, warranting cautious interpretation (http://ihmeuw.org/3o6q). Only with more reliable local data can these estimates be further refined.

Significant progress has been made in the production of ARV and RIG. The abandonment of nerve tissue vaccines has been mitigated by the production of cell culture vaccines, and efforts are ongoing to produce ARV and RIG for human use. Nevertheless, availability and supply of vaccine remains a matter of concern, especially in remote areas where transportation and cold chain maintenance are big challenges. Also, increasing production costs and quality requirements may impede future production of ARV and RIG for human use in the public sector. As it is to be expected that the number of people taking PEP will continue to rise, introduction of cost-effective intradermal rabies vaccination is essential for sustaining the supply of human ARV.

Furthermore, it should be clear that prophylaxis alone is not sufficient to control rabies. Unfortunately, much less success has been made in the formulation and implementation of effective rabies control programmes. Such programmes appear to have been initiated since the late 1970s, yet although some individual projects reported successes, the overall impact has probably been limited due to limited duration and geographical coverage. Most projects seem to have been limited to the Kathmandu Valley, likely because of accessibility, yet available data showed that 94% of all reported dog bites occurred outside of this area. Different state and non-state actors have been involved in rabies control over the years, but collaboration between these different groups has been limited. Illustrative of this is that most projects included awareness and mass dog vaccination, but lacked dog population management activities such as animal birth control or waste management. Prevailing cultural and religious practices should be taken into account when designing dog population management strategies, as for instance the important role of dogs in Hinduism may be an impediment for successful programme adoption [67]. Canine rabies control programmes could further be complemented with deworming against endemic dog-borne parasitic zoonoses such as *Echinococcus granulosus* and *Toxocara canis* [85]. New policy-relevant research, e.g., on the ecology of stray dog populations across the country and the true contribution of the sylvatic cycle, is crucial to develop realistic, long-term control programmes. Finally, with 40% of all rabies occurring in the South Asian region, several regional control efforts are emerging, providing new opportunities for rabies control in Nepal [86].

## Conclusion

Limited data indicate that rabies probably still is a major zoonosis in Nepal. However, more and better data are needed, especially from rural areas, to estimate the true burden of animal and human rabies and to plan, implement and evaluate rabies control programmes. The current control of rabies is hampered by insufficient vaccine availability across the country. The way forward for effective rabies control programmes lies in more collaboration, both within the country and within the region. We believe this disease can be controlled only through a coordinated one health approach [87,88]. To accomplish these recommendations, high-level political commitment is essential. Making rabies the model zoonosis for successful control could be a powerful step towards achieving this.

## Supporting Information

S1 File Search strategy and results: database searches for peer-reviewed and grey literature on rabies in Nepal. (XLSX)

S2 File Search strategy and results: manual searches for peer-reviewed and grey literature on rabies in Nepal. (XLSX)

S3 File The Himalayan Times newspaper articles related to rabies in Nepal, 2010–2014. (DOCX)

S4 File Animal and human data related to rabies in Nepal. (XLSX)

S1 Checklist PRISMA checklist. (DOC)
