# Supplementary material for: Epidemiology, Impact and Control of Rabies in Nepal: A Systematic Review
Source: PLoS Negl Trop Dis. 2016 Feb 12;10(2):e0004461. doi: 10.1371/journal.pntd.0004461 (PMC4752342; doi:10.1371/journal.pntd.0004461)
Supplement: S3 File — (DOCX) [file pntd.0004461.s003.docx]

The Himalayan Times: Rabies Articles

Note: due to updates to the website of The Himalayan Times, the articles listed here were no longer available online at the time of submission (September 2015).

Contents

[20141102—It was incorrect 3](#_Toc428956316)

[20141030—20‚000 stray dogs in KMC 4](#_Toc428956317)

[20141018—13 die of rabies in Jajarkot 5](#_Toc428956318)

[20140922— 6](#_Toc428956319)

[20140910—65yo man killed in fox attack 7](#_Toc428956320)

[20140710—Rabid dogs bite 14 8](#_Toc428956321)

[20140514—Rabid dog terror 9](#_Toc428956322)

[20140511—Rabies terror in Dadeldhura 10](#_Toc428956323)

[20140208—Rabies fears 11](#_Toc428956324)

[20140116—Stray dogs kill minor 12](#_Toc428956325)

[20140116—Flock of stray-dogs notch 5yo boy to death 12](#_Toc428956326)

[20130915—3 people die of rabies in Jajarkot 13](#_Toc428956327)

[20130913—Father‚ daughter die after dog bites in Jajarkot 14](#_Toc428956328)

[20130912—Three die of rabies 15](#_Toc428956329)

[20130718—Street dog terror 16](#_Toc428956330)

[20130523—Cow on the loose attacks 10‚ restrained 17](#_Toc428956331)

[20130304—Adopting strays 18](#_Toc428956332)

[20130201—Rabid dog menace 20](#_Toc428956333)

[20130113—Jackal kills toddler 21](#_Toc428956334)

[20130111—Understanding between two ministries to control zoonosis 22](#_Toc428956335)

[20130108—Rabies terror on in Bahundangi 23](#_Toc428956336)

[20121209—Rabies patients in Siraha visit traditional healers for treatment 24](#_Toc428956337)

[20120815—Single monkey attacks over 100 persons 25](#_Toc428956338)

[20121031—Stray dogs‚ pigs control drive in Gaighat 26](#_Toc428956339)

[20120719—Stray dogs‚ jackals threaten farmers' livelihood 27](#_Toc428956340)

[20120515—Shutdown in Kailali 28](#_Toc428956341)

[20120221—Two die of rabies in Jhapa 29](#_Toc428956342)

[20120123—Don't ignore rabies! 30](#_Toc428956343)

[20120108—Mad dog bites 16 32](#_Toc428956344)

[20110929—'Children at greater risk' 33](#_Toc428956345)

[20110628—Siraha lacks anti-rabies vaccine 34](#_Toc428956346)

[20110427—Rabies awareness drive 35](#_Toc428956347)

[20110413—Anti-rabies drugs crisis at hospital 36](#_Toc428956348)

[20110315—Bharatpur hospital faces anti-rabies vaccine crisis 37](#_Toc428956349)

[20110307—Humane way 38](#_Toc428956350)

[20110307—Number of street dogs 'on decline' 39](#_Toc428956351)

[20110223—Mad dogs scare Manthali folk – Anti-rabies vaccine stocks finished 40](#_Toc428956352)

[20101201—Jackal rampage in Mahottari 41](#_Toc428956353)

[20101023—Shaman claims he can treat rabies with mantras 42](#_Toc428956354)

[20100901—Anti-rabies vaccine crunch hits Rajbiraj 44](#_Toc428956355)

[20100616—Rabies death has villagers panicked 45](#_Toc428956356)

# 20141102—**It was incorrect**

With regard to the news story “20,000 stray dogs in KMC” carried by The Himalayan Times Online on Oct. 30, it has been stated that the “KAT Centre is stopped now”. This is incorrect information.

Kathmandu Animal Treatment Centre was created in 2004 with the aim of managing the street dog population in Kathmandu by sterilization. Since its inception, it has so far sterilized 15,000 stray dogs and is still continuing its work. The centre was never shut down. In fact, it is working more aggressively now than before. We have approached the concerned government authorities several times to support its program but we never receive any kind of help in our fight to control the dog population and its possible spread of rabies in the capital. We would like to request you to correct the above mentioned information and, at the same time, we would also like to request the Kathmandu Municipal authorities to support our programme of controlling the stray dog population.

*Dr. Pushkar Pal, Project Manager, KAT Centre* - See more at: http://thehimalayantimes.com/fullNews.php?headline=LETTERS%3A+Corruption++in+projects&NewsID=432211#sthash.jY60eXNl.dpuf

# 20141030—20‚000 stray dogs in KMC

**KATHMANDU:** Managing the stray cattle and dogs seems to be a real challenge to KMC which is working to fulfil the plan of keeping Kathmandu green and clean.

It is estimated that there are presently 20 thousand stray dogs roaming Kathmandu. Although the Dog House set up some time back by famous comedian duo Madan Krishna Shrestha and Haribansha Acahrya tried to curb the stray dog population through vasectomy, it has stopped now.

Chief of the KMC Police Department, Dhanapati Sapkota, said there is no other way of managing the stray dogs except chasing them away as they cannot be killed due to the animal rights concept. KMC plans to auction off the stray cattle every Sunday.

Last fiscal year, KMC had taken away as many as 388 stray cattle outside Kathmandu and it earned revenue worth Rs 217,000 through auctioning the stray cattle that year. - See more at: http://thehimalayantimes.com/fullNews.php?headline=20%26sbquo%3B000+stray+dogs+in+KMC&NewsID=431863#sthash.vq2GnIwG.dpuf

# 20141018—13 die of rabies in Jajarkot

**JAJARKOT:** As many as 13 people have died of rabies in a very remote Majkot VDC of the district as they depended on shaman for treatment.

According to a local Hari Bahadur Shahi, mad dogs bit people, but they relied on shamanism for treatment instead of taking anti-rabies vaccine.

“After such huge casualties, we are visiting District Health Office for vaccination now. We did blunder by believing in shaman's treatment," he said.

Similarly, more than 60 cattle died of rabies in the locality.

Another local Ratna rued at the District Health Office rued, "I've come for anti-rabies vaccine. Thirteen died so far due to blind belief on shamanism."

It takes two days' walk to reach at Majkot from the district headquarters.

On the other hand, In-Charge at Dashera Health Post, Ram Bahadur Thapa, said although preparations are afoot to manage anti-rabies vaccine at Dashera, they failed to manage it for lack of refrigerator. It forced people to visit the district headquarters. - See more at: http://thehimalayantimes.com/fullNews.php?headline=13+die+of+rabies+in+Jajarkot&NewsID=430916#sthash.MMfdWRaA.dpuf

# 20140922—

**BHAKTAPUR**: On the occasion of the World Rabies Day, the District Livestock Office, Bhaktapur, has initiated a weeklong free anti-rabies campaign.

Office Chief and senior veterinarian Bal Bahadur Chand said the domesticate dogs and cats would be given anti-rabies vaccination from the District Livestock Office and other subordinate offices.

The camp will be organized at different places as at Challing VDC Office today, at Dadhikot VDC tomorrow, and Balkot VDC Office and at Bhaktapur municipality office on September 27.

The campaign was organized to prevent people from getting rabies from cats and dogs.

Similarly, an awareness class will be given for the students from grade 8 to 10 in the community schools.

According to veterinarian Umesh Lal Pradhan, the students in the community schools of Kharipati, Bageshwori, Dadhikot, Sipadol, Nangkhel and Sudal VDCs of the district will be given orientation on how people could remain safe from the rabies virus. - See more at: http://thehimalayantimes.com/fullNews.php?headline=Dharapani+People+bound+to+drink+murky+water&NewsID=428317#sthash.u8xxrw8q.dpuf

# ****20140910—****65yo man killed in fox attack

**DADELDHURA:** An elderly man died of rabies infection in Dadeldhura.

The deceased has been identified as Gagan Tamata (65) of Dugari, Bagarkot VDC-2 of the district.

According to a preliminary investigation, the symptoms of rabies was seen four months after he was bitten by a fox.

According to the District Police Office, Dadeldhura, Tamata died during the course of treatment at the Sub-Regional Hospital.

Meanwhile, normal life in most of the VDC's of the district have been affected due to fox terror.

Police said that two women were critically injured in a fox attack at Sautali, Pipal Chaur VDC of Darchula district.

Tara Devi (55) and Dhana Devi (24) were rushed to the District Hospital Darchula after the incident. - See more at: http://thehimalayantimes.com/fullNews.php?headline=65yo+man+killed+in+fox+attack+&NewsID=426992#sthash.mx9f2KjU.dpuf

# ****20140710—****Rabid dogs bite 14

RAMECHHAP: At least 14 persons were injured after mad dogs bit them at Bamti and Kubhukasthali VDCs in Ramechhap on Thursday. The mad dogs have terrified villagers after rabies spread among dogs in the villages. Fourteen persons have been injured after dogs bit them, said Dr Basudev Pandey of District health Office, Ramechhap. He said anti-rabies vaccines have been sent to affected areas. - See more at: http://thehimalayantimes.com/fullNews.php?headline=ONCE+OVER&NewsID=420791#sthash.M6WrSklZ.dpuf

# 20140514—Rabid dog terror

BAJURA: Villagers of Kotila VDC of Bajura have been terrified after mad dogs bit more than a dozen villagers. Auxiliary Nursing mid-wife Deuma Dhami said all the injured had received anti-rabies vaccines. Locals have been trying to tame the dogs, but to no avail. “We haven’t been able to control the dogs even though we are trying very hard,” said a local. The villagers have urged the administration and police to kill the dogs. — HNS

# 20140511—Rabies terror in Dadeldhura

DADELDHURA: Locals of two remote VDCs of Dadeldhura are terrified that they may contract rabies because they consumed the meat of a goat which was bitten by a jackal.

They had eaten the meat at hotel Poornima in Laldhunga market on May 3.

Likewise, panic has spread among villagers of Bhadrapur VDC of the district after they were informed that they had consumed the milk of a buffalo which was also bitten by the jackal a week ago.

Police arrested the meat seller Bir Singh Mal of Sirsha VDC and the hotel owner Ishowari Datta Joshi today. They have been kept in the custody of district police office.

Suresh Nath, a local, said it was yet to be ascertained how many people had consumed the meat in the hotel.

“A discussion was held in the village regarding the issue yesterday,” he said. He further added that the goat owner had sold 7 kg meat in the village while 10 kg was sold to Poornima Hotel. “I had eaten in the same hotel on April 6,” he said, adding that he received the anti-rabies vaccine at district headquarters Bagkhor because it was scarce in the village.

Meanwhile, officer Kabindra Bista of district public health office said the office has demanded 800 vaccines for Jogbuda and 200 vaccines for Bhadrapur from Dhangadi after the incident came to light.

“We will send the vaccines immediately after they arrive from Dhangadi,” he added. He said the vaccines would be provided after checking consumers’ health.

DSP Bhoj Dev Khatiwada of DPO said action would be taken against the accused if he is proven guilty.

Acting Chief Dharmananda Dhami of the district hospital said as many as 200 villagers had received the vaccine after consuming pork at Sirsha VDC some two months ago. The pig was also bitten by a jackal. - See more at: http://thehimalayantimes.com/fullNews.php?headline=Rabies+terror+in+Dadeldhura&NewsID=414585#sthash.FyHeO8Zw.dpuf

# 20140208—Rabies fears

DIPAYAL: Rabies is spreading fast among cattle in different wards of Doti’s Dauda VDC. According to a local, Ganesh Joshi, the disease has killed eight cattle in course of a week. “Dogs fed on a dead and diseased ox. Rabid dogs bit the cattle, thus the disease spread,” he said, adding the disease had first appeared in Dauda-5 and spread to other villages. - See more at: http://thehimalayantimes.com/fullNews.php?headline=ONCE-OVER&NewsID=405442#sthash.FchTsnjR.dpuf

# 20140116—Stray dogs kill minor

RAUTAHAT: A five-year-old boy was killed on the spot when a pack of stray dogs attacked him at Khesarhiya of Rautahat on Thursday.

Police said Arjun Yadav, son of Sikendra Yadav of the VDC was bitten by a pack of dogs until he died at around 11:00 am. The victim’s grandmother Sarabatiyadevi said the stray dogs attacked the boy when he was defecating in an open area. The entire Khesarhiya village has been terrorised after the shocking incident. The body has been sent to district hospital Gaur for post-mortem. - See more at: <http://thehimalayantimes.com/fullNews.php?headline=ONCE-OVER&NewsID=403200#sthash.qxxDoNJk.dpuf>

# 20140116—Flock of stray-dogs notch 5yo boy to death

RAUTAHAT: Five-year-old Arjun Ray Yadav of Khesarahiya VDC-7 in Rautahat has been notched to death by a flock of stray dogs on Thursday morning.

The only son of local Sikendra Yadav, Arjun was attacked while he had gone out to a nearby field to answer nature’s call.

After hearing little Arjun’s cries, his grandmother went out to rescue him and chased away the dogs, however, it was already late as Arjun had already breathed his last.

Arjun’s dead body has scratches and bruises all over due to dog-attack. His body has been sent to district hospital Gaur for an autopsy, said DSP Dipak Adhikari, In-charge of District Police Office. - See more at: http://thehimalayantimes.com/fullNews.php?headline=Flock+of+stray-dogs+notch+5yo+boy+to+death+&NewsID=403124#sthash.sxHpst61.dpuf

# 20130915—3 people die of rabies in Jajarkot

JAJARKOT: Three people died of rabies infection after being bitten by stray dogs in the district recently.

The deceased are Bal Bahadur Thapa (55) and his daughter Hari Kala Thapa (22) of Dhime VDC-1 and Ratna Bahadur Thapa (48) of Dhime VDC-5.

More than two dozens villagers have been suffering from the disease after being bitten by stray dogs at Dhime and Jagatipur VDCs.

The patients died due to shortage of anti-rabies vaccine at the District Hospital.

The stock of the vaccine has run out and the hospital has demanded for more vaccines from the Regional Health Office, said Dhir Jung Shah, Assistant Chief of the District Public Health Office, Jajarkot. - See more at: http://thehimalayantimes.com/fullNews.php?headline=3+people+die+of+rabies+in+Jajarkot&NewsID=390846#sthash.8zCdvQOe.dpuf

# 20130913—Father‚ daughter die after dog bites in Jajarkot

JAJARKOT: A father and daughter in Jajarkot district died after they were bitten by a rabid dog.

The deceased have been identified as Bal Bahadur Thapa, 55, and his daughter Harikala, 22, of Dhime VDC-1.

They died as they did not take vaccine against rabies. The rabid dog has bitten more than 15 persons in Dhime VDC.

The victims have been facing problems due to lack of vaccine against rabies though they reached the district headquarters searching for the vaccine, it is learnt. - See more at: http://thehimalayantimes.com/fullNews.php?headline=Father%26sbquo%3B+daughter+die+after+dog+bites+in+Jajarkot&NewsID=390643#sthash.SasTrJdQ.dpuf

# 20130912—Three die of rabies

JAJARKOT: Three persons have died and dozens more are infected with rabies virus after being bitten by local dogs in Dhime, Jajarkot. Among the dead are, Harikala Thapa, 22, his father Pal Bahadur, 55, of Batulechowr, Dhime, and a youth from Jajargaun of Dhime. According to police, as many as 15 persons and two dozen cattle have been infected. “We have sent all the infected people to the district hospital for vaccines,” said district livestock office JTA Bheshjung Shah. Lack of anti-rabies vaccine has forced people to travel to Mission Hospital in Chaurjahari, Rukum. - See more at: http://thehimalayantimes.com/fullNews.php?headline=Three+arrested+&NewsID=390619#sthash.IN8eywXx.dpuf

# 20130718—**Street dog terror**

GULMI: Street dogs have unleashed terror in Tamghas of late. Four persons were bitten by street dogs in Tamghas on Thursday. Of them, a seriously injured Kamala Panthi, 44, has been sent to the Capital for treatment after she could not be treated in Tamghas. Local security bodies have notified the public not to walk alone on the road. A meeting of stakeholders at Chief District Officer Kamalraj Dhakal’s office today suggested that dog owners get their pets vaccinated for rabies. Over 10 people have been bitten by dogs in one week. - See more at: http://thehimalayantimes.com/fullNews.php?headline=Murder+in+Siraha+&NewsID=384263#sthash.orislqnm.dpuf

# 20130523—Cow on the loose attacks 10‚ restrained

KATHMANDU: A cow that had been running amok in New Baneshwor since late yesterday was finally taken into control this morning after it attacked 10 people.

Police personnel and Kathmandu Metropolitan City officials got hold of the animal this morning, said Bhim Prasad Dhakal, DSP at the Metropolitan Police Circle, Baneshwor. “The cow attacked 10 people,” said Dhakal.

Police said no one had come to claim the cow till this afternoon, adding that preparations are afoot to send it to Kanji House.

Ramesh Neupane of Baneshwor, who witnessed the incident, said, “It seemed the cow was suffering from rabies.” - See more at: http://thehimalayantimes.com/fullNews.php?headline=Cow+on+the+loose+attacks+10%E2%80%9A+restrained&NewsID=377538#sthash.4IgYrJel.dpuf

# 20130304—Adopting strays

KATHMANDU: All of us have encountered stray dogs in our localiteis and we might have also witnessed them delivering puppies. And usually when the mother is not with its puppies, people tend to take those puppies and keep them as pets. But more often, these puppies grow on the streets and add to the stray population.

However, there is also a darker side to the stray population. Owners take good care of their pets with quality food, love and care. But when these animals suffer from incurable diseases, some of the owners try to get rid of them by leaving them on the streets. And once on the streets, their life of struggles begins — people mistreat them, they are hit by vehicles sometimes leaving them injured and often killing them.

Says Dr Surendra Kumar Basyal, Vet Manager at Animal Nepal, Ekantakuna, “These animals too have the right to live. Their rights shouldn’t be violated. They need care and love from human beings.”

That is the reason why Animal Nepal, a shelter for dogs, cats and oxen came into existence. “The puppies that have lost their mother are adopted and are kept in our shelter at Chobhar and whenever anyone comes with the interest of adopting them, we provide puppies,” Dr Basyal informed.

The stray dogs are also provided treatment here. “The dogs are vaccinated, given calcium and are made ready for adoption,” he said.

Along with care and treatment for these animals, Animal Nepal has also tried to raise public awareness by visiting different schools and informing students about animals. “It has been working for decreasing the number of street animals by controlling the population through birth control methods,” he shared.

Apart from Animal Nepal, there are other organisations too that have been working to change the situation of street animals and Kathmandu Animal Treatment Centre (KAT) is one of them. “We are determined to lessen the number of street dogs in the Valley. As such more than 14,000 dogs have been sterilised by the organisation,” informed Dr Baburam Gautam, one of the board members of KAT.

Also wounded cats and dogs are given treatment in their shelter at Chapali Budhanilkantha. “Animals with different skin diseases are kept in the shelter until they completely recover. Some animals come with fractures in legs and forearms and we keep them in our shelter for treatment,” he revealed.

KAT is also working to raise public awareness for the safety and adoption of dogs and cats.

Cows and bulls are other animals living a miserable life — once the cows stop giving milk, they are left on the streets while the case is worse for bulls as they are left on the streets soon after their birth as they are of no use to people. But Devoted Radical Environmental Animal Movement Society (DREAMS) is working to rescue street bulls and cows. “We are working for the cows and bulls because there is no other organisation working for them. Some of them are hurt in accidents and we provide them treatment. We even file cases against those who hurt animals,” said Prerana Shah, Founder and Treasurer of DREAMS.

They are also working to raise awareness among people who are selling caged birds.

While all three institutions have provision to provide free adoption for the animals in their shelters, there are no such cases where people have approached them for adoption. Though few locals come asking for puppies at Animal Nepal, “we do not have any record of how many have been adopted till date,” said Dr Basyal.

However, if you are willing to adopt animals from these instituitions, you can get them for free. You just need to contact these organisations. - See more at: http://thehimalayantimes.com/fullNews.php?headline=Adopting+strays&NewsID=368213#sthash.EsamU5n2.dpuf

# 20130201—**Rabid dog menace**

KALIKOT: People in two VDCs in Kalikot are living under the fear of rabid dogs. Three people have fallen ill in Lalu and Malkot VDCs after they were bitten by rabid dogs roaming freely in the area. Even cattle in the VDCs have fallen ill after being bitten by stray dogs. The District Livestock Service Office said it has already dispatched the anti-rabies vaccines to the affected areas. Locals said the rabid dogs have still not been killed or taken under control - See more at: http://thehimalayantimes.com/fullNews.php?headline=Youth+found+dead+&NewsID=364370#sthash.C62M5u4D.dpuf

# 20130113—Jackal kills toddler

KATHMANDU: A wild animal thought to be a jackal dragged away and devoured a two-year-old girl in Mahottari, police said today.

The toddler was snatched as she played alone by a sugarcane plantation near her home in the district of Mahottari, bordering India, yesterday and found dead the following morning by locals who said only her head remained intact.

“The body of the child was found on the banks of a river 300 metres from her house on Sunday morning,” said police officer Jaya Narayan Yadav. “The remains of her body looked similar to the remains of a goat eaten by a jackal. The wild animal had eaten the flesh and there was only the remains of the skeleton of the chest and the bones of the legs,” he added.

The Indian or Himalayan jackal, which is similar in appearance to a small wolf and can carry rabies, is abundant in the southern Tarai plains and shelters in holes in dense brush on the outskirts of villages and farms.

It is primarily a scavenger which eats garbage and offal, although it is known to supplement its diet with rodents, reptiles, fruit and insects and does occasionally kill poultry, goats and lambs. - See more at: http://thehimalayantimes.com/fullNews.php?headline=Jackal+kills+toddler&NewsID=361781#sthash.lhszskDA.dpuf

# 20130111—Understanding between two ministries to control zoonosis

KATHMANDU: An understanding was reached between two ministries, Ministry of Health and Population, and Ministry of Agriculture, to move in a joint venture to control zoonosis, animal disease that can be transmitted to humans.

According to World Health Organization (WHO), 60 per cent of the diseases that are seen in human beings are transmitted through animals. Medical experts said the diseases like bird flu, rabies, Japanese encephalitis, and tuberculosis, among others, are transmitted to humans from the animals.

The two ministries have made the understanding to control the zoonosis as per the one -door health policy prepared by the Nepal Veterinary Council. A Health Secretary level agreement on this was signed between India and Nepal a month ago.

Speaking at a programme organised to give information about the one-door policy here today, Jaya Mukunda Khanal, Secretary at the Ministry of Agriculture, stressed further collaboration between the two ministries to control the transmission of Zoonosis.

The One-door Health Policy was brought with the concept that human beings can be healthy only when the animals are healthy, Khanal said and expressed his commitment for the effective implementation of the policy.

Similarly, Chief of the Epidemiology and Disease Control Division of the Department of Health Services, Dr. G.D. Thakur said the two ministries have working unity for the last two years.

Chairman of the Council, Dr. Shuva Narayan Mahato, and Registrar Dr. Narayan Prasad Ghimire said animal health was as important as human health and that the one-door policy was brought by integrating both.

Meanwhile, the council is going to elect a new working committee through election today itself. The Council has 698 veterinary doctors affiliated to it. - See more at: http://thehimalayantimes.com/fullNews.php?headline=Understanding+between+two+ministries+to+control+zoonosis&NewsID=361412#sthash.keuhZk9z.dpuf

# 20130108—Rabies terror on in Bahundangi

BHADRAPUR: Locals of Jhapa's eastern VDC, Bahundangi, have been worried with the outbreak of rabies there for the past one week.

More than half a dozen cattle including cow and buffalo have been so far killed in wards 1,2, 4 and 6 of the VDC due to rabies while it has also affected other livestock and stray dogs.

The rabies outbreak has created a havoc in the VDC after a rabies dog has bitten a domestic cattle in the first week of last December, according to the District Livestock Service Office. - See more at: http://thehimalayantimes.com/fullNews.php?headline=Rabies+terror+on+in+Bahundangi+&NewsID=360962#sthash.EwmM66dG.dpuf

# 20121209—Rabies patients in Siraha visit traditional healers for treatment

SIRAHA: People seeking rabies treatment are turning to traditional healers rather than getting medical help at Gothtole, a Muslim community in Lahan Municipality.

Following the outbreak, health workers have not been seen in the village out of fear of getting infected, locals said.

"We have been forced to see the local shaman after there were no signs of health workers in the village despite our frequent requests," they said.

Tension ran high in the Muslim community after Mohammad Nurahasan, 18, also known as Bhhausha, died of rabies on Wednesday.

According to locals, the shaman uses a dinner plate as a means of treatment. He spells his mantras (spells) after placing the plate on the back of the dog-bitten people.

"If the plate gets stuck on the back, it is confirmed that the person is infected otherwise not," locals said adding that Saturday, Sunday and Wednesday were considered the appropriate day for treatment.

As many as 20 people had the plate stuck on their backs on Saturday, and 32 people on Sunday, said a local, Prem Paswan.

According to him, locals are afraid that they might have been infected and were unaware about the infection as they always hung out with Bhhauaha.

More than 50 people could be infected with rabies, said another local, Mohammad Mustak, adding that they were yet to receive any vaccines or health workers from the District health Office.

The DHO is 40-km away from the village. Locals demanded that they be provided with the vaccines through the Lahan hospital.

As many as 16 youths are learnt to have received the shots from Lahan hospital. - See more at: http://thehimalayantimes.com/fullNews.php?headline=Rabies+patients+in+Siraha+visit++traditional+healers+for+treatment&NewsID=357131#sthash.f8wJ5ap8.dpuf

# 20120815—Single monkey attacks over 100 persons

BARDIBAS: A single monkey has bitten over 100 people in two VDCs in northern part of Mahottari district during the last three days.

Locals near the highway in Bardibas and Gauribas VDCs said that they were at the receiving end of the monkey menace. Sujan Khatiwada, a local at Gauribas-2, said that over 40 persons were hurt in the attack.

Similarly, at least 60 persons were injured at main bazaar area in Bardibas in the attacks of monkeys, said businessman Shambhu Bhattarai.

However, the injured are likely to suffer further as the Bardibas Hospital has no treatment services to the victims. Acting chief of the hospital, Radheshyam Jha, has admitted that it lacks anti-rabies vaccines as the hospital receives only 20 vaccines in a month. Area Police Office, Bardibas, said that the frantic monkey has not been caught yet. - See more at: http://thehimalayantimes.com/fullNews.php?headline=Single+monkey+attacks+over+100+persons&NewsID=343549#sthash.J9TUETny.dpuf

# 20121031—Stray dogs‚ pigs control drive in Gaighat

GAIGHAT: The menace created by stray dogs and pigs in different Wards of the Triyuga Municipality has subsided after the municipality started killing such animals.

The municipal authorities were compelled to launch the campaign of killing such animals after the number of stray dogs and pigs increased drastically in the town and started troubling the people, said Khilaraj Rai, the Chief Executive Officer of Triyuga Municipality.

The Municipality started killing the stray dogs and pigs by giving them poisoned meat and fodder.

The population of stray dogs and pigs shot up especially in Gaighat Bazaar, Bokse Bazaar, Jaljale, Deuri Bazaar, Rajabas and Chuhade areas in the town. - See more at: http://thehimalayantimes.com/fullNews.php?headline=Stray+dogs%26sbquo%3B+pigs+control+drive+in+Gaighat+&NewsID=352610#sthash.C6nna5xB.dpuf

# 20120719—Stray dogs‚ jackals threaten farmers' livelihood

MYAGDI: Stray dogs and jackals killed at least 300 Himalayan goats in upper Mustang, making farmers suffer huge loss.

Nima Dhinduk Gurung, a farmer, said that over 300 Himalayan goats were killed in Chhoser VDC in the last three months after stray dogs and jackals attacked them. Gurung said the stray dogs and jackals chase the goats and kill them in the pasture land when locals take the goats for grazing.

Gurung said new-born goats are the main prey of these predators. “At least seven goats have been killed from each house. We have been compelled to rethink on the best alternative to goat rearing due to the terror of stray dogs and jackals,” he said.

Goat rearing is the main occupation as well as the main source of income for the people of upper Mustang. Gurung said that they were facing difficulty controlling the stray dogs and jackals as killing them with poison would have an adverse effect on snow leopards. The stray dogs and jackals have even attacked horses and cows.

Nima Tsering Gurung of Kagbeni said the stray dogs and jackals have terrorised farmers in Chhonhup, Lho-Manthang, Surkhang, Chhusang and Kagbeni too. The farmers have expressed dissatisfaction towards lack of seriousness on the part of the District Livestock Office against this menace.

However, veterinary technician Aita Bahadur Thakali said the office was ready to extend technical support to farmers if the locals formally sought assistances. - See more at: http://thehimalayantimes.com/fullNews.php?headline=Stray+dogs%26sbquo%3B+jackals+threaten+farmers%27+livelihood&NewsID=340238#sthash.RQJuQ5z2.dpuf

# 20120515—Shutdown in Kailali

KAILALI: Patients have been hit hard due to bandh in Kailali, officials said today.

“Every day over 50 people come to get rabies vaccines shot here but due to regular bandh, we are unable to administer the vaccines,” said Dhangadhi-based Seti Zonal Hospital emergency ward In-charge Krishna Bohara.

Meanwhile, agitators at the Dadeldhura headquarters Baghkhor intercepted and handed over to the police an ambulance belonging to a primary health centre of Deukhel VDC, Bajhang that was carrying liquor bottles, vegetables and diesel while returning after taking a patient to Dhangadhi.

According to the police, the agitators had intercepted the vehicle, while checking it at the bus park. “Agitators would have torched the ambulance vehicle had we not reached the spot on time,” said DSP Binod Ghimire, adding the police would not leave the driver anytime soon though will leave the ambulance following the due process.

“We will take serious action against the ambulance driver,” said DSP Ghimire. Different demonstrations were taken out in Kailali, Kanchanpur and Bajura with the ongoing far-west agitation entering its 18th day today. - See more at: http://thehimalayantimes.com/fullNews.php?headline=Shutdown+in+Kailali&NewsID=332035#sthash.ZkZ2u90H.dpuf

# 20120221—Two die of rabies in Jhapa

JHAPA: Two persons have died of rabies at Mechinagar-10 Kakadvitta in Jhapa district during a week.

The death of two locals due to rabies has terrorised the entire community.

Rohan Joshi, 42, of Purano Bhansar of Kakadvitta died on Monday while undergoing treatment in Siliguri, India.

North Bengal Medical Hospital in Siliguri confirmed that Joshi was suffering from rabies.

Similarly, Serina Shrestha, 7, of Bahundangi Road of Kakadvitta also died of rabies.

She was rushed to Kathmandu after the BP Koirala Institute of Health Sciences, Dharan, referred her to Kathmandu for further treatment, saying that she was suffering from rabies.

It is said that dog had bitten the deceased some four months ago.

It is learnt that both of them were not administered anti-rabies vaccines.

Local Gopal Siwakoti said over 200 rabid dogs have been straying in different areas of Kakadvitta for long. - See more at: http://thehimalayantimes.com/fullNews.php?headline=Two+die+of+rabies+in+Jhapa&NewsID=321335#sthash.rlN2ZFcy.dpuf

# 20120123—Don't ignore rabies!

KATHMANDU: There are many animals, some are your pets while others are stray animals. However there are chances, you can be attacked by them, exposed to their saliva or scratched by them. This is dangerous because that animal can be a rabid animal, which is a carrier of rabies virus.

Physician Dr Buddha Karki, Bir Hospital said, “Any animal bite is infectious either from a dog, cat, rabbit, monkey, bat, rat, et cetera because every animal’s mouth is filled with bacteria. It can infect a human body if entered through bitten skin, eyes, mouth or nose. Moreover, rabies is a viral infection, which is developed when exposed to rabid animals — salvia, bites and scrape.”

The carriers

There are two types of animals carrying high and low possibilities of transferring rabies to human beings. Among them bat, dog, fox, wild dog and wild cat carry higher chances of transmitting rabies, however cat, monkey, rat, rabbit and squirrel are of low risk category. Dr Karki said, “In the US the most common rabies carrier animal is bat while in our country and worldwide dog bite is the major cause of rabies. However, rabies vaccines prevent from developing the disease.” Rabies is a viral infection that is preventable. However, it is usually fatal if it is untimely vaccinated.

“Symptoms of rabies may develop within 10 days after exposure if not vaccinated timely. But the symptoms may not show immediately after exposure, it can take two months or years,” said Dr Karki.

It can get fatal

When a rabid animal bites you, virus content saliva of that animal, inserts into your body. Also the virus has greater chances of entering human body when infected animal’s saliva is in contact with your eyes, nose or mouth.

“These viruses replicate rapidly in human body and attack nerve cells quickly so it is also called neurotropic virus. When multiplied virus attacks, it causes an inflammation in the brain and brain fails to function properly. Patient suffers from anxiety, has swallowing difficulties, paralysis and at last the patient dies,” informed Dr Karki.

Immediate treatment

Whenever a stray dog bites you, it is difficult to find whether it is normal or rabid bite. Dr Karki said, “In such condition one must seek immediate vaccination. But, if your vaccinated pet bites you, you are not at risk. Moreover, if a bat bites, touches you or even if it hovers around, you need to go for immediate vaccination because bats are reservoir of rabies.”

Cat bites are also harmful. It also demands medical attention. However, according to Dr Karki, “Monkey, cat and rat bites and scratches are rare and have low risk of transmitting rabies; but, immediate treatment is a must.”

“Immediately after exposure, you must clean the area with soap water and apply antibiotics, get tetanus vaccine within 24 hours and one must get rabies vaccination within 48 hours. In addition, it is better to leave the wound open to avoid developing infection,” said Dr Karki. If the animal bite is in sensitive parts like face and above torso, immediately immunoglobulin medicine is given.

Pre and post vaccination

There is an easy accessibility of rabies vaccines in every health care centre near you. The post exposure anti-rabies vaccine of five doses protects from developing infection.

“At the mean time, there is an availability pre-exposure vaccine with three doses, which works for three years. The vaccine is recommended for people in endemic area, slum areas with risk of dog bite and people in veterinary services,” added Dr Karki. If you have pets in your house, vaccination of rabies timely is pertinent. - See more at: http://thehimalayantimes.com/fullNews.php?headline=Don%27t+ignore+rabies%21&NewsID=317811#sthash.h36Grba5.dpuf

20120108—Mad dog bites 16

BIRATNAGAR: A rabies-infected dog on Sunday bit 16 persons at Jamunagachhi, Saruchiya and Janapathtole before locals thrashed the animal to death. Of the bite victims, 10 have been administered vaccine against rabies at Koshi Zonal Hospital while the rest received the vaccine at private medical centres. Most of the victims are students and government employees. Sources at Koshi Zonal Hospital said they administered anti-rabies vaccine to 35 dog bite victims from Biratnagar and its adjoining areas. - See more at: http://thehimalayantimes.com/fullNews.php?headline=Two+killed%26sbquo%3B+scores+hurt&NewsID=315810#sthash.Ua9uVSQK.dpuf

# 20110929—'Children at greater risk'

KATHMANDU: World Rabies Day was observed today with various programmes to raise awareness and understanding about rabies, the oldest and deadliest disease known to humankind.

Khageshwar Sharma, director at Himalayan Animal Rescue Trust said children were at higher risk of contracting rabies than adults. He said, “Children are often unaware that dogs transmit rabies and may not tell their parents when they are bitten, licked or scratched by an animal infected with the disease.”

He further said that awareness was the best defence against rabies. Dr Russell Lyon, volunteer veterinarian at the trust said, “Vaccinating domestic animals is the single most important way of preventing the spread of rabies.”

Veterinarians say that rabies is a viral disease that can be transmitted from animals to humans mainly when animals bite, but the disease can also be contracted through exposure to saliva of an infected animal.

According to World Health Organisation, around 55,000 people die every year from rabies — half of them are children under the age of 15. - See more at: http://thehimalayantimes.com/fullNews.php?headline=%27Children+at+greater+risk%27&NewsID=304272#sthash.bOVkevtj.dpuf

# 20110628—Siraha lacks anti-rabies vaccine

SIRAHA: Patients with rabies have been deprived of treatment as the District Hospital, Siraha, is reeling under shortage of anti-rabies vaccine

The district hospital daily receives a flow of 10-15 patients bitten by rabid dogs. But they couldn’t receive treatment because of lack of anti-rabies vaccine, said hospital chief Dr Daya Shankar Karna

According to Karna, the government hasn’t made anti-rabies vaccine available as per the demand. Siraha alone needs around 5,000 anti-rabies vaccine sets every year. However, the government is providing only 3,000 sets, he said

Since anti-rabies vaccine is expensive in private hospitals and clinics, many patients have to visit the government-owners hospitals for treatment.

- See more at: http://thehimalayantimes.com/fullNews.php?headline=Siraha+lacks+anti-rabies+vaccine+&NewsID=293490#sthash.eGXyhDh8.dpuf

# 20110427—Rabies awareness drive

KATHMANDU: Nepal Veterinary Association (NVA) is marking World Veterinary Day with an awareness campaign on rabies with the global theme “One World One Health: Rabies Eradication Our Goal”.

An NVA press statement said it would observe a week-long programme to mark the day starting April 24.

According to WHO, more than 55,000 people die of rabies every year and 40 per cent bitten by suspect rabid animals are children under 15. Dogs are the source of 99 per cent of human rabies deaths, according to WHO.

The statement said around 200 people die of rabies annually in the country. Epidemiology and Disease Control Division provides vaccines to around 30,000 people annually. It has recorded 1,692 deaths from 2000 to 2009 due to dog and fox bite. There are around 35,000 stray dogs in Kathmandu Valley alone.

NVA has urged the concerned bodies to act to solve the problem of stray dogs and to make anti-rabies vaccine available.

NVA further urged dissemination of information about rabies. - See more at: http://thehimalayantimes.com/fullNews.php?headline=Rabies+awareness+drive&NewsID=285738#sthash.Tgu9jpxP.dpuf

# 20110413—Anti-rabies drugs crisis at hospital

JANAKPURDHAM: Patients visiting Janakpur Zonal Hospital hospital have been hit hard due to shortage of anti-rabies vaccines there. The hospital has not received any consignment of the vaccine in the past two weeks.

The government provides anti-rabies vaccine free of cost at government hospitals, but patients have been compelled to pay much more for the same at private hospitals and clinics.

Patients said the hospital administration has shown no interest in bringing additional vaccines.

The locals of five districts including Dhanusha visit the hospital to treat rabies.

According to Pradip Raya Yadav, pharmacy inspector at the hospital, the hospital ran out of stock before the expected time due to increased number of rabies patients. He added, ‘’The patients are now compelled to purchase the vaccine on their own as the hospital does not have it.’’

He informed that the hospital receives 80 vaccines per month, but said suggested that to meet the demand of patients, the hospital needs 200 vaccines every month. Shyamkrishna Karki of Dhanushadham-1 , Dhanusha lamented that he was compelled to buy the vaccine from the market at a high price and that commoners had been hit hard as the government had failed to provide the necessary vaccines to the hospital. ‘’The hospital administration and the related authority should solve the problem at the earliest,’’ said Karki. - See more at: http://thehimalayantimes.com/fullNews.php?headline=Anti-rabies+drugs+crisis+at+hospital&NewsID=283789#sthash.OWEic7cP.dpuf

# 20110315—Bharatpur hospital faces anti-rabies vaccine crisis

CHITWAN: Bharatpur Hospital faces a shortage of anti-rabies vaccine by 30-35 percent every month.

The hospital receives 250 vaccines every month despite an increasing flow of patients. Mainly patients from the poor and backward community visit the hospital.

At present the hospital can provide service to only 60 patients where 100 rabies patients visit in an average, said Dr. Jagannath Tiwari of the hospital. Each rabid patient needs five vaccines for full dose.

Tiwari said the hospital is dealing with the problem by buying extra vaccines on its own cutting the budget that the hospital receives in other titles. Still some patients have to buy from other private hospitals and medical centers, he said. A rabies vaccine costs Rs. 600 in Chitwan.

The hospital has informed the Department of Epidemiology about the problem. An official of the department, Latnarayan Shah, said the demand for anti-rabies vaccine will be addressed soon.

- See more at: http://thehimalayantimes.com/fullNews.php?headline=Bharatpur+hospital+faces+anti-rabies+vaccine+crisis&NewsID=280059#sthash.GHDtKME5.dpuf

# 20110307—Humane way

To learn that the number of our “best friends” on the Kathmandu city streets have declined, may evoke mixed feelings for the inhabitants. By all accounts, as per a report by the Kathmandu Animal Treatment Centre (KAT Centre), through a city-wide survey inside the Ring Road, the stray dog population has declined to 22,500 by September 2010, from the earlier 31,000. The decline is attributed to sterlisation of the female dogs. Earlier, the method had been to eliminate the stray dogs, which was quite cruel and crossed the boundary of animal rights. The present drive is more humane, and also helps in checking the transmission of rabies from dogs to human beings.

In the country, rabies results in the deaths of scores of people every year. The deaths could have been averted had timely anti-rabies injection been administered to the person bitten by an infected dog.. Therefore, the sterilisation drive must continue not only in the capital but in all the parts of the country so that the dog population is kept under control. Besides this, the street dogs in general must be given anti-rabies vaccination as a lasting solution. - See more at: http://thehimalayantimes.com/fullNews.php?headline=Setting+it+right&NewsID=279081#sthash.esT5az6Y.dpuf

# 20110307—Number of street dogs 'on decline'

KATHMANDU: The number of dogs in urban Kathmandu has significantly decreased, with the dog population being expected to sharply decline in the next generation, a recent report based on a city-wide survey of the street dog population of the Metropolitan city has revealed.

The report released by Kathmandu Animal Treatment Centre (KAT Centre) said the number of street dogs in urban Kathmandu has decreased from around 31,000 to approximately 22,500 and the percentage of sterilised female dogs increased from 15 per cent to 40 per cent leading to a likely decline in the number of dogs.

The KAT Centre and the government had conducted the survey on all dogs living on the streets inside the Ring Road of Kathmandu, Nepal in September 2010.

The KAT Centre is a charitable organisation working with its state goal of reducing the stray dog population of the Kathmandu Valley and eliminate rabies through Animal Birth Control (ABC) and rabies vaccinations.

- See more at: http://thehimalayantimes.com/fullNews.php?headline=Number+of+street+dogs+%27on+decline%27&NewsID=279049#sthash.uJzt0wE2.dpuf

# 20110223—Mad dogs scare Manthali folk – Anti-rabies vaccine stocks finished

MANTHALI: Rabid dogs have been causing panic among local people of Ramechhap district headquarters Manthali and Gothgaun VDC, among others, for a week.

In course of a week, mad dogs have bitten more than 200 pedestrians in the district.

Locals fears’ have accentuated after Biraj Shrestha of Gothgaun-9 died of the disease some days ago.

According to DSP Prem Bahadur Karki, the disease has affected Manthali and Gothgaun VDC the most. Since Monday, stray dogs have bitten 24 pedestrians in Manthali, 19 of them in a single day.

The terror-stricken people like Januka Karki hesitate to send their children to school, even as Dr Narayan Shrestha of the District Veterinary Office says the office has initiated a campaign to terminate the rabid dogs.

Dr Shrestha concedes that the disease has spread in Gothgaun, Gelu, Tilpung, Rampur, Phulasi VDCs apart from Manthali. According to Binda Karki of Gothgaun, mad dogs have bitten more than 70 people in her village in the last few days.

Grappling with the canine terror, the district is in dire straits, thanks to a shortage of anti-rabies vaccines.

“In the last few days, we have administered anti-rabies vaccine to 190 people. We have run out of vaccines and are waiting for fresh supplies,” says medical officer of the Manthali Primary Health Centre, Ramhari Subedi. - See more at: <http://thehimalayantimes.com/fullNews.php?headline=Mad+dogs+scare+Manthali+folk&NewsID=277536#sthash.jYS3xNpo.dpuf>

# 20101201—Jackal rampage in Mahottari

MAHOTTARI: Jackal rampage has been on the rise in northern village of the district while shortage of medicines in the health posts has hit hard locals seeking service.

Jackals have terrorized villagers in Belgachhi Village Development Committee (VDC), ward 5 and 6 for the past two days by biting into cattle and humans. As of today, locals including Ram Kumari Devi, Atma Saha, Pinki Kumari, Sangeeta Thapa have become the victims of jackal attack.

Similarly, jackal sank its teeth into a cow of Tahale Balampaki and goat of Ramshrista Saha, informed Bhola Mahato, a local.

Victims, who reached health post Gaushala for treatment after jackal bite, were compelled to return home with empty hand due to the lack of immune against rabies, said ram Kumari Devi.

- See more at: http://thehimalayantimes.com/fullNews.php?headline=Jackal+rampage+in+Mahottari+&NewsID=267754#sthash.7j5K1U0m.dpuf

# 20101023—Shaman claims he can treat rabies with mantras

Though science refutes, locals' faith in the tantrik is so huge they keep thronging his house

SIRAHA: Siraha district vice chairman of Madheshi Janadhikar Forum-Loktantrik Bechan Mahato set out on early Saturday morning to Lalpur VDC-8 to visit tantrik Baue Lal Singh. Mahato was accompanied by his wife and two sons. The reason behind his reaching there was none other than to seek treatment for his dog-bitten sons by the witch doctor.

Mahato seemed to have been firmly believing that his sons could be cured by the tantrik treatment of Baue Lal. “I know well that anti-rabies vaccine should be injected after dog-bite,” said Mahato. “However, I am also convinced that the vaccine will not be required after Baue Lal casts his mantras on them.”

Mahato is just a case in point. Baue Lal is a popular shaman here and is visited by locals from various parts of the district, especially seeking treatment after a dog-bite, in a way, seeking treatment for rabies.

Baue Lal (55) is a well known personality in the area and people believe that he can treat through his spells even diseases like rabies. Not surprisingly, he has been practicing the conventional treatment for over 40 years.

Around 10 people bitten by dogs visit Baue Lal’s house every day. He, however, considers Sunday and Wednesday to be the appropriate day for treatment.

Baue Lal uses a silver plate and soil collected by mouse as the means of treatment. He spells his mantras after placing the silver plate smeared with the soil on the back of the dog-bitten people. “If the plate gets stuck on the back, it is confirmed that the poison has entered the body through wounds, otherwise not,” says Baue Lal.

If he finds that the wound has absorbed the poison, he prescribes herbal roots and only then does he spell the mantras. “If the poison is detected, it necessitates the mantras for up to five weeks depending on the nature of patients,” says Baue Lal.

Claiming that he has a cent percent successful rate in treating dog-bite, the witch doctor says, “I have treated around 10,000 dog-bitten people and not a single patient has died of the rabies.”

Having been involved in the tantrik treatment for over 40 years, Baue Lal, however, does not receive any fees for the treatment. Instead, he appeals to them to feed sages and sanyasis.

A local social worker Bal Krishna Gautam says patients frequent to Baue Lal’s house, as they have to bear huge cost and hurdles to visit hospitals. “Commoners don’t get easily available anti-rabies vaccines and are forced to face various obstacles,” said Gautam. “So, people rather find it easy to visit him.” Gautam suggests that the government launch awareness campaign. According to Gautam, the government must raise awareness among locals that dog-bitten people should visit hospitals. He said anti-rabies vaccines must be made easily available.

Dr Dilip Kumar Jha, former medical officer at Lahan Hospital, does not agree that rabies can be cured through tantrik spells. “The case is that rabies doesn’t get transferred by the bite of healthy dogs. If the animal is infected with rabies, the tantrik treatment cannot prevent the infection,” said Dr Jha.

Dr Jha added that there is no guarantee that a person could be saved even by medical treatment if s/he is infected with rabies virus, as science has not yet discovered medicine for this, let alone the mere traditional tantrik treatment. “Therefore, it is wise to be inoculated with anti-rabies vaccine,” suggested Dr Jha. - See more at: http://thehimalayantimes.com/fullNews.php?headline=Shaman+claims+he+can+treat+rabies+with+mantras&NewsID=262776#sthash.YFaI4Z79.dpuf

# 20100901—Anti-rabies vaccine crunch hits Rajbiraj

RAJBIRAJ: Increase in dog bite cases and a chronic shortage of anti-rabies vaccines at the Sagarmatha Zonal Hospital has local people mad, literally.

At the root of the problem is the Health Department’s inability to meet the rising demand for vaccines. Nonetheless, hospital staffers have been facing local people’s wrath over the shortage.

Hospital chief Dr Laxman Mandal said, ‘’The department sends only 300 anti-rabies vaccines monthly, while the number of those seeking the vaccines exceeds five hundred a month.’’

Mandal went on to say that he had written to the department seeking fresh supplies, but to no avail. He added that the hospital staffers had been finding it hard to deal with angry visitors. Vaccine prices range from Rs 500 to Rs 3,000.

Locals claimed that the department sends 4-500 vaccines in other districts, but it sends only 300 vaccines to the hospital in Saptari, which has a high population density. - See more at: http://thehimalayantimes.com/fullNews.php?headline=Anti-rabies+vaccine+crunch+hits+Rajbiraj&NewsID=256145#sthash.qwSiScfS.dpuf

# 20100616—Rabies death has villagers panicked

ITAHARI: The death of a fellow villager from rabies has terrified people of Sunsari district’s Singiya VDC ward no 3 and 4.

Nandalal Choudhari of Singiya-3 died of rabies on June 7 after the Dharan-based BP Koirala Institute of Health Sciences and Inaruwa-based District Hospital and turned him back home, saying they had run out of the vaccine.

Ramesh Choudhari of Singiya said the villagers, who had shared food, cigarettes and come into Chaudhary’s contact were further terrified after the district hospital sent them back, citing the vaccine shortage.

Local health workers suspect more than 20 villagers, including Ramesh Choudhari, Chanalidevi, Dinesh, Ramitadevi, Gunjadevi and Ritadevi, might have contracted the disease.

Locals have been pressing the local health post to make efforts to get the vaccine. - See more at: http://thehimalayantimes.com/fullNews.php?headline=Rabies+death+has+villagers+panicked+&NewsID=247133#sthash.I2iIvFmN.dpuf
